# Repurposing fusidic acid as an antimicrobial against enterococci with a low probability of resistance development

**DOI:** 10.1007/s10123-024-00506-w

**Published:** 2024-03-27

**Authors:** Mark M. Abdelmassih, Maha M. Ismail, Mona T. Kashef, Tamer Essam

**Affiliations:** Department of Microbiology and Immunology, Faculty of Pharmacy, Cairo University, Cairo, 11562 Egypt

**Keywords:** *Enterococcus faecalis*, *Enterococcus faecium*, Fusidic acid, Repurposing, Treatment, Vancomycin-resistant enterococci

## Abstract

Drug repurposing constitutes a strategy to combat antimicrobial resistance, by using agents with known safety, pharmacokinetics, and pharmacodynamics. Previous studies have implemented new fusidic acid (FA) front-loading-dose regimens, allowing higher serum levels than those achievable with ordinary doses. As susceptibility breakpoints are affected by serum level, we evaluated the repurposing of FA as an antimicrobial product against enterococci. FA minimum inhibitory concentrations (MICs) against standard enterococci strains; *Enterococcus faecalis* ATCC 29212 and *Enterococcus faecium* ATCC 27270 were 2 and 4 µg/mL, respectively. The MIC against 98 enterococcal clinical isolates was ≤ 8 µg/mL; all would be susceptible if categorized according to recalculated breakpoints (≥ 16 µg/mL), based on the serum level achieved using the front-loading regimen. FA administration in vivo, using the BALB/c mouse infection model, significantly reduced bacterial burden by two to three log_10_ units in the liver and spleen of mice infected with vancomycin-susceptible and -resistant strains. Exposure of the standard enterococcal strains to increasing, but not fixed, FA concentrations resulted in resistant strains (MIC = 128 µg/mL), with thicker cell walls and slower growth rates. Only one mutation (M651I) was detected in the *fus*A gene of the resistant strain derived from serial passage of *E. faecium* ATCC 27270, which was retained in the revertant strain after passage in the FA-free medium. In conclusion, FA can be repurposed as an antimicrobial drug against enterococci with a low probability of mutational resistance development, and can be employed for treatment of infections attributable to vancomycin-resistant enterococci.

**Supplementary Information:**

The online version contains supplementary material available at 10.1007/s10123-024-00506-w.

## Introduction

Multidrug-resistant enterococci are challenging nosocomial pathogens that have developed resistance to most antimicrobials in use. Vancomycin-resistant enterococci are classified by the World Health Organization as a high priority pathogen for research and development of new antibiotics (World Health Organization 2017) and by the Centers for Disease Control and Prevention (CDC) as a serious threat. In addition, the CDC reported a 16% increase in the rate of vancomycin-resistant enterococci infections since 2019, which may be attributed to the weakened immunity occurring during the COVID-19 pandemic, and associated higher hospitalization rates (Centers for Disease Control and Prevention 2022).

Drug repurposing offers a fast, relatively inexpensive alternative to development of new antimicrobials by repositioning already-known agents alone or in combination with other antibiotics, with established safety, pharmacokinetic, and pharmacodynamic profiles (Dubey et al. 2020).

Fusidic acid (FA) is a bacteriostatic agent that is used for the treatment of *Staphylococcus aureus* infections; it acts through inhibition of protein synthesis by binding the elongation factor G-GDP (EF-G-GDP), preventing peptide translocation and disassembly of ribosomes (Fernandes 2016).

*Enterococcus faecalis* and *Enterococcus faecium* are intrinsically resistant to FA (Clinical and Laboratory Standards Institute 2023; EUCAST 2022). However, new FA front-loading-dose regimens have been suggested (Bulitta et al. 2013; Fernandes 2016), and have succeeded in reaching high *C*_max_ levels (140–250 µg/mL). The achieved high plasma levels of FA decrease its clearance from the body and lessen the emergence of resistant strains during the treatment period (Kusanya et al. 2011; Tsuji et al. 2011; Bulitta et al. 2013).

Here, we evaluated the repurposing of FA as an antimicrobial agent against enterococci, and whether its use will be accompanied by resistance development.

## Materials and methods

### Bacterial strains and culture conditions

Bacterial strains used or developed in this study are described in Table 1. In addition, 98 clinical enterococcal isolates from the archive of the Microbiology and Immunology department, Faculty of Pharmacy, British University in Egypt, were used in the study. The clinical isolates were randomly obtained from urine samples of patients with urinary tract infections; their antimicrobial susceptibility was unknown when retrieved for this study purpose. All strains were stored in brain heart infusion (BHI) broth (Oxoid, England) containing 25% glycerol at − 80 °C (master seed lot). When required, samples from the master seed lot were cultured on BHI agar (Oxoid, England), at 37 °C for 24 h.

**Table 1:** Bacterial strains used in this study

Strain	Characteristics	Source
*Enterococcus faecalis* ATCC 29212	Standard strain	Dr. Yomna Hashem
*Enterococcus faecium* ATCC 27270	Standard strain	Dr. Samar Mabrouk
*E. faecalis* strain-74	Vancomycin-sensitive	BUE archive
*E. faecalis* strain-Y3	Vancomycin-resistant	BUE archive
*E. faecalis* cP15Q	*E. faecalis* ATCC 29212 passaged in 0.25 of the FA MIC^1^	Current study
*E. faecalis* cP15H	*E. faecalis* ATCC 29212 passaged in 0.5 of FA MIC^1^	Current study
*E. faecalis* cP15Qf	*E. faecalis* cP15Q passaged in FA-free MHB^2^	Current study
*E. faecalis* cP15Hf	*E. faecalis* cP15H passaged in FA-free MHB^2^	Current study
*E. faecalis* iP15	*E. faecalis* ATCC 29212 passaged in increasing concentrations of FA^1^	Current study
*E. faecalis* iP15f	*E. faecalis* iP15 passaged in FA-free MHB^2^	Current study
*E. faecium* cP15Q	*E. faecium* ATCC 27270 passaged in 0.25 of FA MIC^1^	Current study
*E. faecium* cP15H	*E. faecium* ATCC 27270 passaged in 0.5 of FA MIC^1^	Current study
*E. faecium* cP15Qf	*E. faecium* cP15Q passaged in FA-free MHB^2^	Current study
*E. faecium* cP15Hf	*E. faecium* cP15H passaged in FA-free MHB^2^	Current study
*E. faecium* iP15	*E. faecium* ATCC 27270 passaged in increasing concentrations of FA^1^	Current study
*E. faecium* iP15f	*E. faecium* iP15 passaged in FA-free MHB^2^	Current study

*BUE* British University in Egypt, *FA* fusidic acid, *MHB* Muller Hinton broth, *MIC* minimum inhibitory concentration

^1^Passaged serially during 15 days

^2^Passaged consecutively for 10 days

### Identification of clinical enterococci

Clinical enterococcal isolates were identified to species level by PCR targeting the d-alanine-d-alanine ligase (*ddl*) gene according to Dutka-Malen et al. (1995).

### Determination of FA minimum inhibitory concentration and minimum bactericidal concentration

The minimum inhibitory concentration (MIC) of FA against the enterococcal standard strains (*E. faecalis* ATCC 29212 and *E. faecium* ATCC 27270), as well as the clinical isolates, was determined using broth microdilution according to CLSI guidelines (Clinical and Laboratory Standards Institute 2018). FA was prepared as a stock solution; different dilutions were made in Muller-Hinton (MH) broth to achieve a concentration range from 128 to 0.25 µg/mL. Inoculated and uninoculated MH broth were used as positive and negative controls, respectively. The experiment was done in triplicate.

The minimum bactericidal concentration (MBC) was determined as described by Rodríguez et al. (2022) for the standard enterococci. At the end of the incubation period of MIC plates, 100 μL of the wells showing no visible growth were inoculated onto BHI agar followed by incubation overnight at 37 °C. The MBC corresponds to the minimum concentration of FA reducing the initial inoculum (5 × 10^5^ CFU/mL) by > 99.99% (absence of visible colonies on the agar surface). The experiment was done in triplicate.

### Calculation of estimated FA susceptibility breakpoint

The FA susceptibility breakpoint was estimated in a trial to determine whether the elevated *C*_max_ recorded previously when applying the front-loading dose (two front loading doses of 1650 mg every 12 h followed by 13 doses of 825 mg every 12 h) compared to that recorded when using the ordinary dose regimen (500 mg of oral FA) would theoretically affect the FA breakpoint. The simple equation previously adopted by the British Society for Antimicrobial Chemotherapy (BSAC) for breakpoint calculation [(*C*_max_/et) × fs] was used where *C*_max_ is the maximum serum concentration after administering a stated dose at steady state, *f* is a protein binding factor, *t* is the half-life of serum elimination, *e* is the minimal *C*_max_:MIC ratio (usually 4), and *s* is the reproducibility. In the case of FA, *f* was 0.2, *t* was 0.5, and *s* was 0.5 or, better, 1 (MacGowan and Wise 2001). When using the ordinary dose regimen (500 mg of oral FA), the *C*_max_ was about 30 µg/mL, while the *C*_max_ was 157 and 256 µg/mL, after the last loading and maintenance doses, respectively, using the front-loading-dose regimen (Bulitta et al. 2013; Fernandes 2016).

### Determination of the antimicrobial susceptibility pattern

The susceptibility of the clinical enterococcal isolates to different antimicrobials was determined by the Kirby-Bauer disk diffusion method (Clinical and Laboratory Standards Institute 2015). The following antibiotics were tested: chloramphenicol (30 μg), minocycline (30 μg), vancomycin (30 μg), ciprofloxacin (5 μg), fosfomycin (200 μg with 50 μg glucose 6-phosphate), nitrofurantoin (300 μg), and linezolid (10 μg). All antibiotic discs were from Oxoid, Basingstoke, Hampshire, UK, except the fosfomycin discs which were from Mast Co., Merseyside, UK. The plates were incubated at 37 °C for 24 h, the inhibition zone diameters were measured, and the results interpreted according to CLSI guidelines (Clinical and Laboratory Standards Institute 2023b). *E. faecalis* ATCC 29212 was used as a control strain.

### In silico detectability of transmissible FA resistance genes in enterococcal genomes 

The detectability of transmissible genes responsible for FA resistance (*fus*B, *fus*C, *fus*D) was determined in the genomes of *E. faecalis* (*n* ≈ 3000) and *E. faecium* (*n* ≈ 5000) publicly available in the Pathosystems Resource Integration Center (PATRIC) database and in all available genomes within the National Center for Biotechnology Information (NCBI). The genomes were searched for the presence of annotated *fus*B, *fus*C, or *fus*D genes. Detectability of gene sequences of *fus*B (accession number NG_047900.1), *fus*C (accession number NG_050413.1), and *fus*D (accession number NG_050585.1) in the genomes of the tested *Enterococcus* spp. also was evaluated using the blastn tool in the PATRIC and NCBI databases.

### In vivo evaluation of the FA efficiency in the protection against *E. faecalis* infection

The in vivo infection model was performed using female BALB/c mice aged 6–8 weeks, weighing 18–22 g (obtained from Theodor Bilharz Research Institute, Giza, Egypt). Mice were housed at room temperature with an alternating 12 h light–dark cycle, fed on standard food and water ad libitum. They were allowed to acclimatize for four days before the start of the experiment, and were observed to exclude any local or systemic diseases (Yin et al. 2015).

#### Establishment of FA dosing regimen

Administration of FA in mice is highly challenging due to its rapid metabolism and short half-life that leads to rapid clearance from the body (Garcia Chavez et al. 2021). To establish the treatment dose and administration route of FA, a pilot study was performed. Mice were divided into groups; each was injected with a different dose of sodium fusidate (375, 180, 90, 60, 45, 30 mg/kg body weight q8h) either by intraperitoneal or by subcutaneous routes (2 mice/dose in each group) and observed for 96 h for survival or any signs of illness. The maximum tolerated dose was used in the in vivo model.

#### *E. faecalis* infection model and FA administration

Neutropenia was induced in 30 female BALB/c mice by intraperitoneal administration of cyclophosphamide (GLS PHARMA Limited, New Delhi, Delhi, India) in volumes of 200 µL, at doses of 150 mg/kg and 100 mg/kg on days 1 and 4, respectively (Onyeji et al. 2000). On day 3, mice were divided into five groups each consisting of six mice: two positive control groups (group 1 and group 3), two FA-treatment groups (group 2 and group 4), and one negative control (group 5). The FA-treatment groups received 200 µL of 60 mg/kg body weight of sodium fusidate in sterile water (equivalent to 57.6 mg/kg body weight FA) intraperitoneally q8h. The positive control groups were administered sterile water instead of sodium fusidate. A negative control group was injected with 200 µL of sterile water intraperitoneally. FA administration continued till the end of the experiment. On day 5, infection was performed by *E. faecalis* strain-74 (vancomycin-susceptible strain) and *E. faecalis* strain-Y3 (vancomycin-resistant strain) by intraperitoneal administration of 10^9^ CFU/mL of either strain suspended in 1 mL saline (Ali et al. 2019). Each strain was used to infect one positive control and one treatment group (Figure S1). The negative control group was administered 1 mL of sterile saline intraperitoneally.

On day 6 (one-day post-infection), all mice were anaesthetized with an overdose of sodium thiopental and then decapitated. The liver and spleen were aseptically dissected and homogenized in sterile phosphate-buffered saline. The viable bacterial count in the dissected organs was determined by tenfold serial dilution of the organ homogenates in phosphate-buffered saline and spotting 10 µL of the undiluted homogenates and of each dilution on the surface of bile esculin azide agar (Biokar Diagnostics, Allonne, Oise, France). Plates were incubated overnight at 37 °C and the enterococcal colonies counted at the end of the incubation period (Zhang et al. 2023).

### Evaluation of possible resistance development to FA

Possible resistance development by repeated exposure to FA was tested by passage of the tested strains (*E. faecalis* ATCC 29212 and *E. faecium* ATCC 27270) in fixed (0.25 and 0.5 of the MIC) and increasing (1 to 256 µg/mL) FA concentrations for 15 days, followed by passage in a FA-free medium for 10 days (Navarro et al. 2019; Ibrahim et al. 2023). The inoculum was prepared by adjusting the optical density (OD) of the overnight bacterial culture in MH broth to 0.5 McFarland unit. The OD-adjusted culture was diluted 1:150 with fresh MH broth (≈ 1 × 10^6^ CFU/mL). The diluted bacterial culture (1 mL) was mixed with 1 mL of FA-containing MH broth to reach the desired FA concentration and incubated at 37 °C overnight. After incubation, the resulting culture was adjusted to an OD equivalent to 0.5 McFarland unit, diluted, and mixed with FA-containing broth as described earlier. In case of passage with increasing FA concentrations, the culture from the well with the highest FA concentration showing visible bacterial growth was used. Passage was repeated daily for 15 consecutive days. After the 15th passage, the resulting cultures were isolated on bile esculin azide agar, and their MIC was determined in triplicate by broth microdilution. During each passage, inoculation of the same strain in a FA-free MH broth was used as a positive control. Uninoculated MH broth was used as a negative control.

The resulting strains, after the 15th passage, were subjected to ten consecutive passages in FA-free MH broth. The obtained cultures after the 10th passage were isolated and their MICs were determined as described previously. The resulting strains are described in Table 1.

### Evaluation of potential resistance mechanisms in the developed resistant strains

Phenotypic and genotypic changes associated with emergence of FA resistance were compared in the parent strains (*E. faecalis* ATCC 29212 and *E. faecium* ATCC 27270), the resistant strains obtained after passage in increasing FA concentrations (*E. faecalis* iP15 and *E. faecium* iP15), and the revertant strains after passage in FA-free MH broth (*E. faecalis* iP15f and *E. faecium* iP15f).

#### Growth pattern determination

Isolated colonies of the tested strains on BHI agar were inoculated into 5 mL BHI broth and incubated overnight at 37 °C with shaking at 180 rpm. The overnight cultures were diluted with BHI broth to reach an OD of 1 at 600 nm, followed by 1/100 dilution in BHI broth. The diluted cultures were incubated at 37 °C with shaking at 180 rpm and the OD of the incubated cultures at 600 nm was measured hourly for 10 h then after 24 h (Tag ElDein et al. 2021). The growth rates were determined according to Tsuchiya and colleagues (Tsuchiya et al. 2018). The experiment was done in triplicate.

#### Antimicrobial susceptibility to different antibiotics

The antimicrobial susceptibility was determined as described previously by the Kirby-Bauer disk diffusion method.

#### Measurement of cell wall thickness

The thickness of the cell wall of the tested strains was measured by transmission electron microscopy (TEM) in the TEM laboratory, Faculty of Agriculture, Cairo University Research Park, Giza, Egypt. The tested strains were grown overnight in BHI broth at 37 °C and 180 rpm and the resulting cultures diluted as described under growth pattern determination. The diluted culture was incubated at 37 °C and 180 rpm until reaching mid-log phase (OD_600_ ≈ 0.8–1). Cultures were centrifuged at 6037 × *g* for 5 min and the pellets were fixed, stained, and scanned by TEM (Nakamura et al. 2021). For each sample, 5 cells were examined by measuring the cell wall thickness at three different locations of each cell.

#### Detection of mutations in *fus*A and *fus*E genes

##### Primer design

Possible mutations in *fus*A and *fus*E genes encoding EF-G and ribosomal protein L6 (a contact area for EF-G), respectively, were determined in resistant and revertant strains and compared to those genes in the parent strains. The nucleotide sequences of *fus*A and *fus*E genes and their flanking regions in *E. faecalis* ATCC 29212 (accession number CP008816.1) were downloaded from the NCBI and the primers were designed to amplify the complete sequence of each gene: FusA-F1 (5′ GGCAGCAGAAAGTCAATT3′) and FusA-Rv1 (5′ATCATCATAAGCTATTTTACACGAA3 ′) for *fus*A, and FusE-F1 (5′ CTGACAAAGAAGCTAGAGC3′) and FusE-Rv1 (5′ TGCTTGTTGTTGACTTCTG 3′) for *fus*E.

The nucleotide sequences of *fus*A and *fus*E genes of *E. faecium* ATCC 27270 were not available in the NCBI database at the time of experiment; so the nucleotide sequences of the aforementioned genes in different *E. faecium* strains (*n* = 9) were downloaded from the NCBI (Table S1) and aligned using Multiple Sequence Alignment (Clustal Omega) tools (https://www.ebi.ac.uk/Tools/msa/clustalo/). Primers were designed in the conserved regions flanking each gene: FusA-F3 (5′ GACCTCAGATAGTGTCCTTAT3′) and FusA-Rv2 (5′ACCACATGCCCATATCATC3 ′) for *fus*A, and FusE-F2 (5′ CTGATAAAGAAGCTCGTGA3′) and FusE-Rv2 (5′ AGGTAAACTTCAGCCAAATG 3′) for *fus*E.

Additional primers FusA-F2 (5′ TCACTGGAATCATTGACTTG3′) and fusA-F4 (5′ CACAGGAATTATCGACCTTG3′) were designed to be used during the sequencing of *fus*A genes from *E. faecalis* and *E. faecium*, respectively (*fus*A gene lengths = 2082 and 2088 bp). All primers were manufactured by Macrogen Inc. (Seoul, South Korea).

##### Amplification of target genes

DNA was extracted from the tested strains using the boiling method (Queipo-Ortuño et al. 2008). For resistant strains, we failed to extract the DNA by the boiling method; the DNeasy blood and tissue kit (Qiagen, Hilden, Germany) was used according to the manufacturer’s instructions.

Amplification of the target genes was performed using PCR in a 50-µL reaction volume containing 10 µL of extracted DNA (2 µL of DNA in case of *E. faecalis* iP15 and *E. faecium* iP15 strains), 10 µL colorless GoTaq® Flexi Buffer (5 ×) (Promega, Madison, Wisconsin, USA), 2 mM magnesium chloride (Promega), 0.2 mM each of nucleotide mix (Promega), 1.25 U GoTaq® DNA Polymerase (Promega), and 30 pmol of each of the forward and reverse primers. The reaction included an initial denaturation step at 95 °C for 3 min followed by 30 cycles of denaturation for 30 s at 95 °C, annealing for 30 s at 52 °C, and extension for 3 min at 72 °C, with a final extension step at 72 °C for 10 min.

##### Sequencing of amplified genes

The amplified gene products were purified using FavoPrep™ Gel/PCR Purification Mini Kit (Favorgen Biotech Corp., National Biotechnology Park, Ping Tung, Taiwan) according to the manufacturer’s recommendation. The purified products were sequenced by Macrogen Inc. using the standard Sanger sequencing method. Two forward primers and the reverse primer were used for sequencing the *fus*A gene (FusA-F1, FusA-F2, and FusA-Rv1 for *E. faecalis* strains; FusA-F3, FusA-F4, and FusA-Rv2 for *E. faecium* strains). For *fus*E gene sequencing, the forward primers were used. The obtained sequences of the *fus*A gene (3 contigs, each contig ≈ 700 bp) were assembled manually. *fus*A and *fus*E gene sequences were translated using the ExPASy translation tool (https://web.expasy.org/translate/) and aligned using Multiple Sequence Alignment (Clustal Omega) tools (https://www.ebi.ac.uk/Tools/msa/clustalo/) to detect possible mutations in the translated peptide sequences.

### Statistical analysis

Statistical analysis was performed using GraphPad Prism 6 (GraphPad Software Inc., CA, USA). All experiments were performed in triplicate and the results were expressed as means ± standard deviation. Student’s *t*-test with Welch’s correction was used to compare the difference in the bacterial load in treatment and control groups. One-way ANOVA with Tukey’s post hoc multiple comparison test was used to assess the difference in cell wall thickness measured by TEM and the growth rate. In addition, two-way ANOVA with Tukey’s post hoc multiple comparison test was used to compare the OD of bacterial cultures at different time points. In all cases, *p* < 0.05 was considered statistically significant.

## Results

### Identification and antimicrobial susceptibility pattern of the clinical isolates

The clinical enterococcal isolates were identified to species level by PCR targeting the *ddl* gene. Only seven isolates were *E. faecium*, while the remaining isolates (*n* = 91) were *E. faecalis*. All enterococcal isolates were susceptible to linezolid and nitrofurantoin, and most were susceptible to fosfomycin (98.9%) and vancomycin (97.9%). About half of the isolates were susceptible to ciprofloxacin (55.1%) and chloramphenicol (53%), while only 6.2% were susceptible to minocycline.

### FA susceptibility in different strains

The MIC of FA against *E. faecalis* ATCC 29212 and *E. faecium* ATCC 27270 was 2 µg/mL and 4 µg/mL, respectively. The MBC values for both strains were 128 µg/mL. Most of the clinical isolates (66/98, 67%) had FA MICs of 2 µg/mL; the remaining isolates had a MIC between 0.5 and 8 µg/mL, yielding an MIC_90_ of 4 µg/mL. The results of typing, susceptibility to different antimicrobials, and FA MIC of each isolate are given in Table S2.

The estimated FA breakpoint, calculated according to *C*_max_ obtained using the ordinary dose regimen, was 1.5 or 3 µg/mL, depending on the *s* value used. Calculated FA breakpoints, according to the *C*_max_ obtained with the front-loading dose regimen, were between 7.85 and 15.7, and between 12.8 and 25.6 µg/mL using the recorded *C*_max_ after the last loading and maintenance doses (≥ 8 and ≥ 16 µg/mL, respectively). The MIC recorded for *E. faecalis* ATCC 29212 and *E. faecium* ATCC 27270, as well as all the clinical isolates tested here, will be less than the estimated breakpoint (can be clinically susceptible to FA using the front-loading-dose regimen). Only two clinical isolates (2%) would be classified as resistant according to the estimated breakpoint using the recorded *C*_max_ after the second dose; these two isolates would be categorized as susceptible if the *C*_max_ value obtained after subsequent doses were applied.

### Transmissible FA resistance in enterococci

No transmissible FA resistance genes (*fus*B, *fus*C, or *Fus*D), were detectable in the publicly available genomes of *E. faecalis* or *E. faecium*.

### In vivo efficiency of FA in the protection against *E. faecalis* infection

**Fig. 1:**
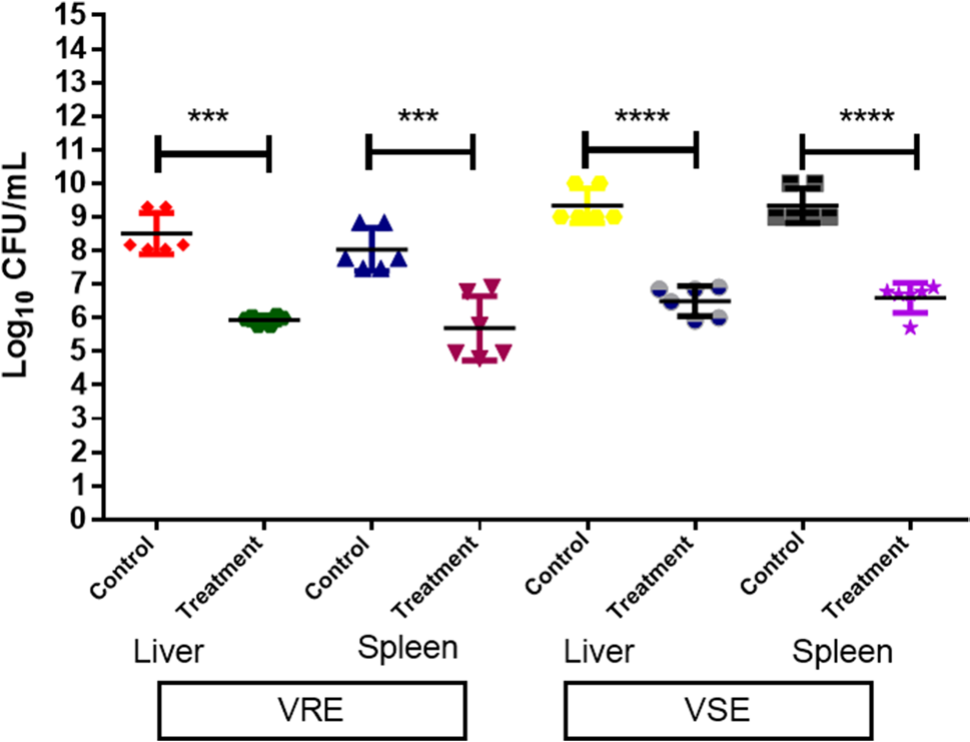
Efficiency of fusidic acid to protect against *Enterococcus faecalis* infection. The viable microbial counts of vancomycin-sensitive *E. faecalis* (VSE) and vancomycin-resistant *E. faecalis* (VRE) retrieved from the liver and spleen of fusidic acid–treated mice were lower by 2–3 log_10_ units compared to those of untreated (positive control) mice. Statistical analyses were performed using Student’s *t*-test with Welch’s correction. ***p < 0.001, ****p < 0.0001; Error bars represent the standard deviation

The in vivo efficiency of FA to protect against enterococcal infection was evaluated in a mouse model. A pilot study to determine the maximum tolerated dose and route of administration of FA indicated that intraperitoneal administration of 60 mg/kg body weight q8h was well-tolerated. Administration of sodium fusidate significantly reduced bacterial colonization by two to three log_10_ units in the liver and spleen of mice infected with either vancomycin-sensitive or vancomycin-resistant enterococci (*p* < 0.001; Fig. 1).

### Development of resistance by exposure to FA

**Fig. 2:**
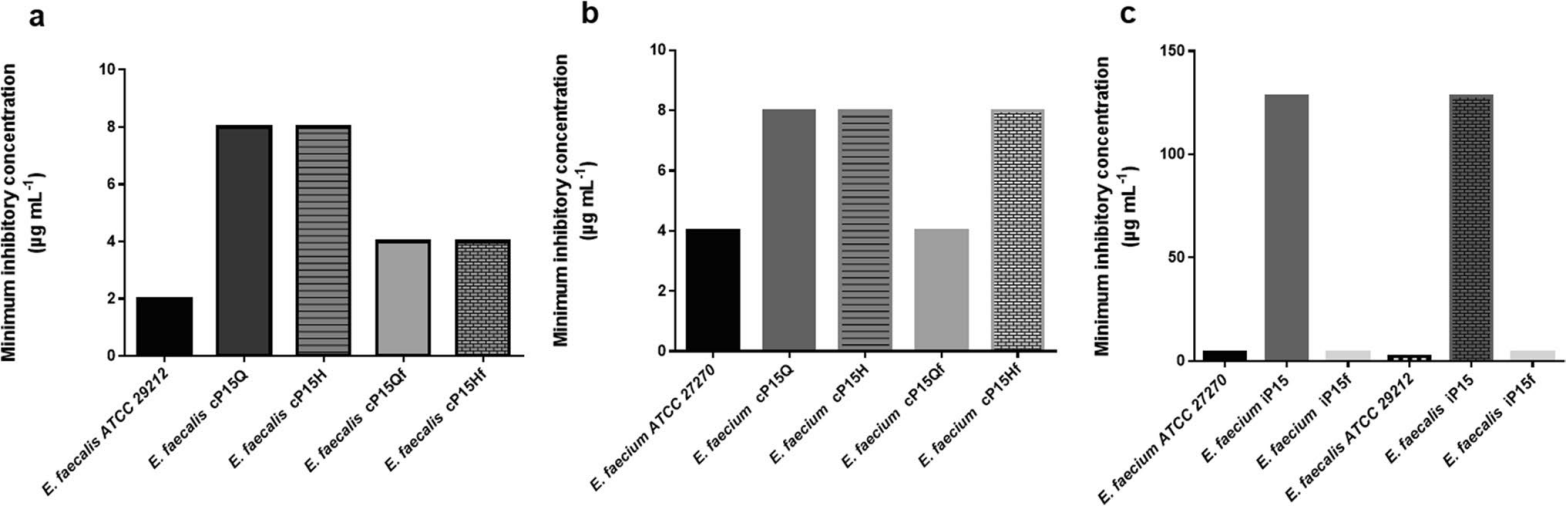
Resistance development to fusidic acid by repeated exposure. **a** The minimum inhibitory concentration (MIC) of *Enterococcus faecalis* ATCC 29212, the resultant strains (*E. faecalis* cP15Q, *E. faecalis* cP15H) after exposure to fixed fusidic acid concentrations (0.25 and 0.5 of the MIC, respectively) for 15 days, and the recovered strains after passage in fusidic acid–free Muller—Hinton (MH) broth for 10 days (*E. faecalis* cP15Qf, *E. faecalis* cP15Hf, respectively); **b** the MIC of *Enterococcus faecium* ATCC 27270, the resultant stains (*E. faecium* cP15Q, *E. faecium* cP15H) after exposure to fixed fusidic acid concentrations (0.25 and 0.5 of the MIC) for 15 days, and the recovered strains after subsequent passage in fusidic acid–free MH broth for 10 days (*E. faecium* cP15Qf, *E. faecium* cP15Hf, respectively); **c** the MIC of *E. faecalis* ATCC 29212, *E. faecium* ATCC 27270, the resultant strains after passage in increasing concentrations of fusidic acid for 15 days (*E. faecalis* iP15 and *E. faecium* iP15, respectively), and the revertant strains after subsequent passage in fusidic acid–free MH broth for 10 days (*E. faecalis* iP15f and *E. faecium* iP15f)

*E. faecalis* ATCC 29212 and *E. faecium* ATCC 27270 were exposed to subinhibitory FA concentrations (0.25–0.5 MIC) for 15 consecutive passages. All the resulting strains (*E. faecalis* cP15Q, *E. faecalis* cP15H, *E. faecium* cP15Q, and *E. faecium* cP15H) by the fifteenth passage had a FA MIC of 8 µg/mL. Subsequent passage of the resultant strains in FA-free MH broth reduced the MIC in *E. faecalis* cP15Qf, *E. faecalis* cP15Hf, and *E. faecium* cP15Qf to 4 µg/mL; *E. faecium* cP15Hf that had been exposed to 0.5 MIC of FA retained its MIC of 8 µg/mL (Fig. 2a, b).

Exposure of *E. faecalis* ATCC 29212 and *E. faecium* ATCC 27270 to increasing concentrations of FA during 15 serial passages resulted in emergence of strains with a MIC of 128 µg/mL (*E. faecalis* iP15 and *E. faecium* iP15). Serial passage of these resistant strains in FA-free medium for 10 days reduced the MIC of the revertant strains (*E. faecalis* iP15f and *E. faecium* iP15f) to 4 µg/mL (Fig. 2c).

### Phenotypic and genotypic changes associated with FA resistance

#### Growth patterns of the developed strains

**Fig. 3:**
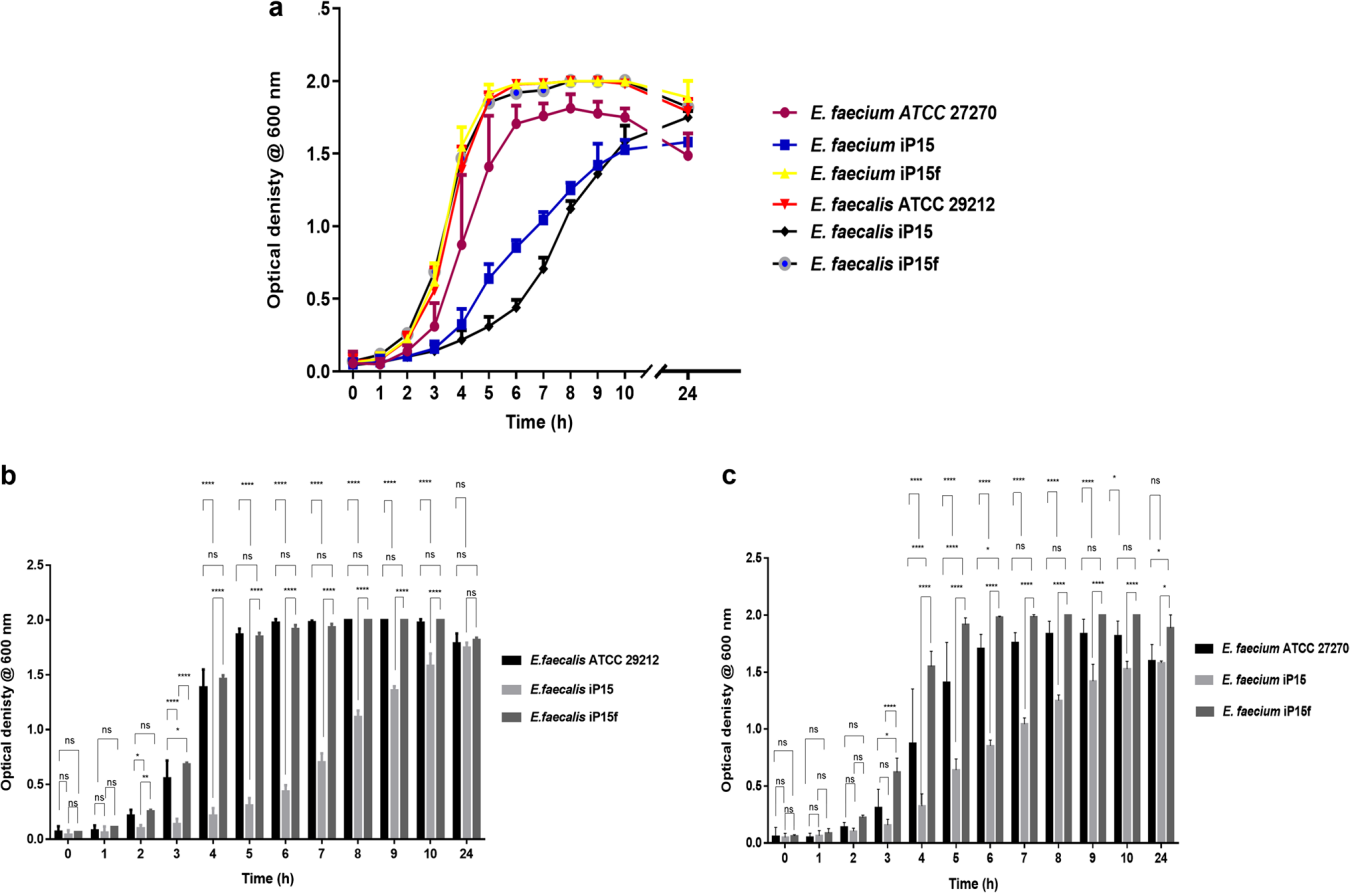
Growth patterns of the parent, resistant, and revertant strains. **a** Growth patterns of the parental *Enterococcus faecalis* ATCC 29212 and *Enterococcus faecium* ATCC 27270, resultant resistant strains after exposure to increasing fusidic acid concentrations (*E. faecalis* iP15, *E. faecium* iP15), and revertant strains after passage of the resistant strains in fusidic acid–free medium (*E. faecalis* iP15f, *E. faecium* iP15f). The optical density was measured hourly at 600 nm for 10 h then after 24 h; **b** OD_600_ of the cultures of *Enterococcus faecalis* ATCC 29212, *E. faecalis* iP15, and *E. faecalis* iP15f was compared at each time point using two-way ANOVA with Tukey’s post hoc multiple comparison test; **c** OD_600_ of the cultures of *Enterococcus faecium* ATCC 27270, *E. faecium* iP15, and *E. faecium* iP15f was compared at each time point using two-way ANOVA with Tukey’s post hoc multiple comparison test. *p < 0.05, **p < 0.01, ***p < 0.001, ****p < 0.0001; ns, non-significant. Error bars represent the standard error

The resistant strains exhibited slower growth compared to the parent and the revertant strains (Fig. 3a), during the exponential growth phase. The OD_600_ of the cultures was compared at different time points, where the resistant *E. faecalis* iP15 had significantly reduced growth during the period from 2 to 10 h and reached a similar OD after 24 h of incubation, compared to the parent *E. faecalis* ATCC 29212 and the revertant *E. faecalis* iP15f (*p* < 0.05; Fig. 3b). Similarly, the resistant *E. faecium* iP15 showed a significantly reduced growth between 3 and 10 h of incubation and recorded similar growth after 24 h of incubation, compared to the parent *E. faecium* ATCC 27270 and the revertant *E. faecium* iP15f (*p* < 0.05; Fig. 3c). The growth rate of the resistant strains (*E. faecalis* iP15 and *E. faecium* iP15) were significantly lower than those of the parent strains (*E. faecalis* ATCC 29212 and *E. faecium* ATCC 27270; *p* < 0.01) and the revertant strains (*E. faecalis* iP15f and *E. faecium* iP15f; *p* < 0.05) as indicated in Table 2.

**Table 2:** Mean growth rates of the parent, resistant, and revertant strains

Mean growth rate ± standard deviation (h^−1^)					
*E. faecium* ATCC 27270	*E. faecium* iP15	*E. faecium* iP15f	*E. faecalis* ATCC 29212	*E. faecalis* iP15	*E. faecalis* iP15
0.7252 ± 0.0174^a^	0.6108 ± 0.0431^b^	0.7174 ± 0.0215^a^	0.7252 ± 0.0751^c^	0.3751 ± 0.114^d^	0.6592 ± 0.020^c^

Mean values with unlike superscript letters within the same species are significantly different

#### Antimicrobial susceptibility

All the tested strains (the parental enterococci, the resistant strains, and the mutant strains) had the same antimicrobial susceptibility pattern; all were susceptible to the tested antimicrobials (chloramphenicol, vancomycin, ciprofloxacin, fosfomycin, nitrofurantoin, and linezolid).

#### Cell wall thickness

**Fig. 4:**
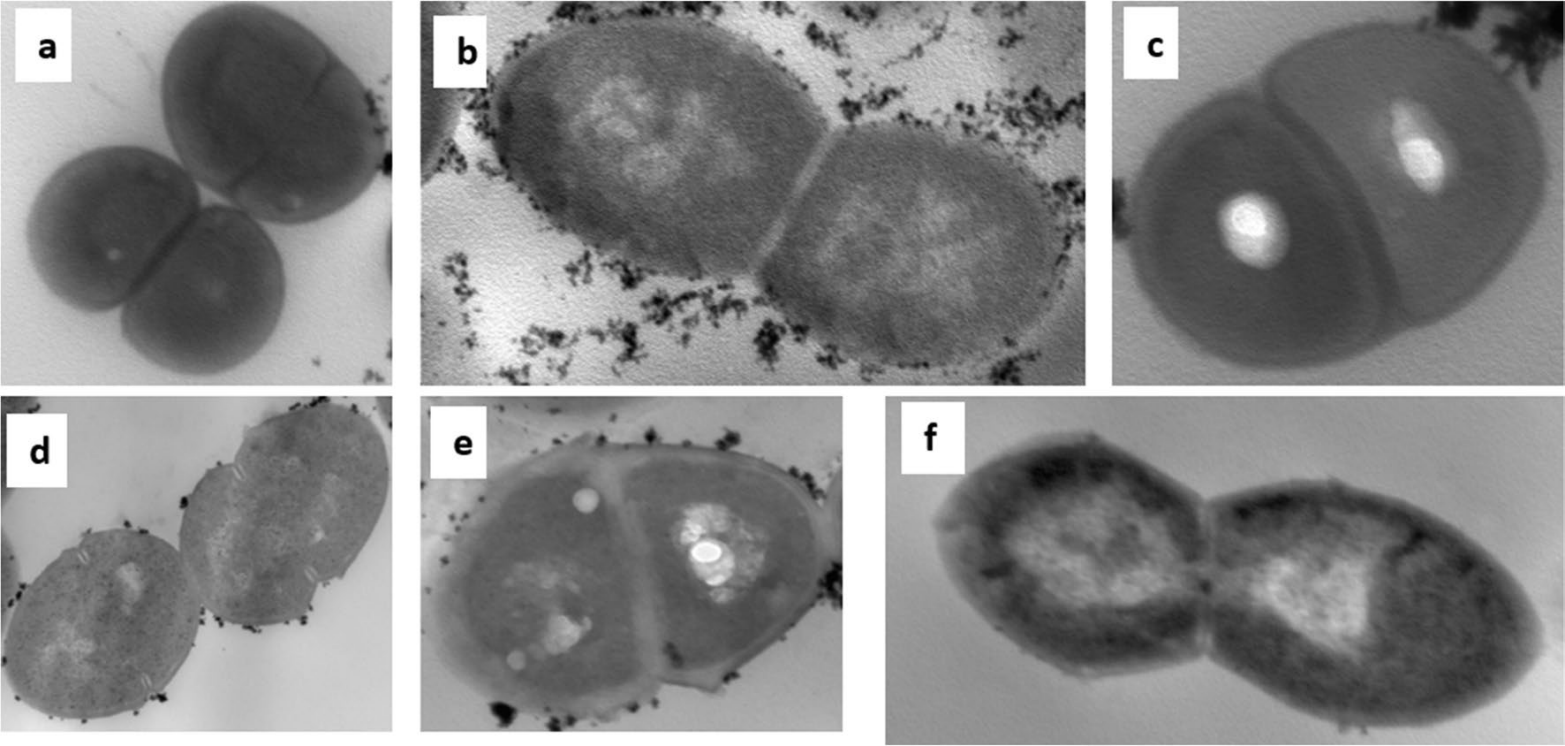
Transmission electron microscopy (TEM) images. TEM micrographs at 40 000 × magnification of **a** parent *Enterococcus faecalis* ATCC 29212; **b** resistant *E. faecalis* iP15 after exposure of *E. faecalis* ATCC 29212 to increasing fusidic acid concentrations during 15 serial passages; **c** revertant *E. faecalis* iP15f after passage of *E. faecalis* iP15 in a fusidic acid–free medium for 10 consecutive passages; **d** parent *Enterococcus faecium* ATCC 27270; **e** resistant *E. faecium* iP15 after exposure of *E. faecium* ATCC 27270 to increasing fusidic acid concentrations during 15 serial passages; and **f** revertant *E. faecium* iP15f after passage of *E. faecium* iP15 in a fusidic acid–free medium for 10 consecutive passages

**Fig. 5:**
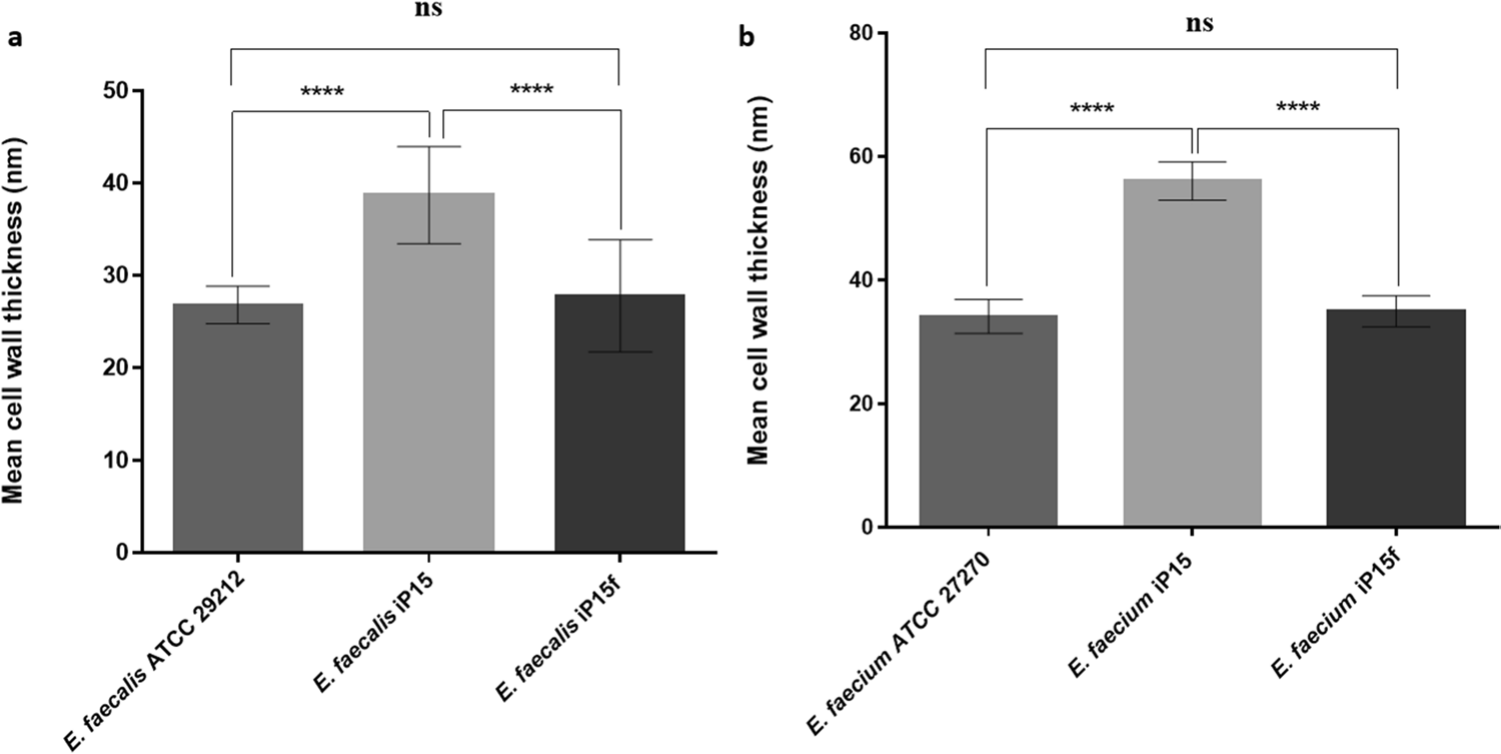
Cell wall thickness measured by the transmission electron microscopy. **a** The cell wall thickness of the parent *Enterococcus faecalis* ATCC 29212, the resistant *E. faecalis* iP15 after exposure of *E. faecalis* ATCC 29212 to increasing fusidic acid concentrations during 15 serial passages, and the revertant *E. faecalis* iP15f after passage of *E. faecalis* iP15 in fusidic acid–free medium for 10 consecutive passages. **b** The cell wall thickness of the parent *Enterococcus faecium* ATCC 27270, the resistant *E. faecium* iP15 after exposure of *E. faecium* ATCC 27270 to increasing fusidic acid concentrations during 15 serial passages, and the revertant *E. faecium* iP15f after passage of *E. faecium* iP15 in fusidic acid–free medium for 10 consecutive passages. All data represent the mean of 15 measurements (five cells with 3 different locations at each cell); error bars represent the standard error of means. Statistical analysis was done using one-way ANOVA with Tukey’s post hoc multiple comparison test. ****p < 0.0001; ns, non-significant

Cell wall thickness of tested strains was measured by TEM (Fig. 4). The resistant strains (*E. faecalis* iP15 and *E. faecium* iP15) had a significantly thicker cell wall (38.73 ± 1.36 and 56.03 ± 0.79 nm, respectively) compared to that of the parent strains, i.e., *E. faecalis* ATCC 29212 and *E. faecium* ATCC 27270 (26.85 ± 0.52 and 34.10 ± 0.71 nm, respectively; *p* < 0.0001), and the revertant strains, i.e., *E. faecalis* iP15f and *E. faecium* iP15f (27.85 ± 1.57 and 34.91 ± 0.65 nm, respectively; *p* < 0.0001) (Fig. 5).

#### Mutations in *fus*A and *fus*E genes

No mutations were detected in the nucleotide or the translated peptide sequences of either *fus*A or *fus*E genes of the resistant *E. faecalis* iP15 or revertant *E. faecalis* iP15f, compared to the parental strain. Only one amino acid change, M651I, was identified in the translated peptide sequence of the *fus*A gene of the resistant *E. faecium* iP15 compared to parental *E. faecium* ATCC 27270; this change was retained in the translated peptide sequence of the *fus*A gene of the revertant *E. faecium* iP15f strain (Figure S2). No mutations were detectable in the sequences of the translated *fus*E genes of *E. faecium* strains. All sequences have been deposited in the Genebank under accession numbers OP686491 (*fus*A, *E. faecalis* ATCC 29212), OP686492 (*fus*A, *E. faecalis* iP15), OR113384 (*fus*A, *E. faecalis* iP15f), OP686493 (*fus*E, *E. faecalis* ATCC 29212), OP686494 (*fus*E, *E. faecalis* iP15), OR113385 (*fus*E, *E. faecalis* iP15f), OP686495 (*fus*A, *E. faecium* ATCC 27270), OP686496 (*fus*A, *E. faecium *iP15), OR113386 (*fus*A, *E. faecium* iP15f), OP686497 (*fus*E, *E. faecium* ATCC 27270), OP686498 (*fus*E, *E. faecium* iP15), and OR113387 (*fus*A, *E. faecium* iP15f).

## Discussion

Most of the identified clinical isolates were *E. faecalis* (91 out of 98), consistent with *E. faecalis* being a more common cause of infection than *E. faecium* (Hashem et al. 2017; Alsanie et al. 2019). The tested clinical isolates had a susceptibility profile similar to that reported for enterococci worldwide (Alsanie et al. 2019; Kateete et al. 2019; Boccella et al. 2021; Coombs et al. 2022; Aung et al. 2023).

The MICs of FA against *E. faecalis* ATCC 29212 and *E. faecium* ATCC 27270 were 2 and 4 µg/mL, respectively. Similar MIC values of FA were reported previously against enterococci (Jones et al. 2011; Fernandes 2016). The FA MBC was 128 ug/mL, indicating its bacteriostatic action on enterococci (Rolston et al. 2017).

FA MICs were further determined for clinical enterococcal isolates, since FA is used frequently in Egypt for treatment of skin and wound infections (Abadallah et al. 2007). The MIC_90_ was 4 µg/mL, and none of the tested isolates had a FA MIC > 8 µg/mL. A similar MIC_90_ value was reported for enterococcal strains (Jones et al. 2011; Fernandes 2016).

The estimated FA susceptibility breakpoint (based on *C*_max_ obtained using ordinary dose regimen) was 1.5 or 3 µg/mL, consistent with the current categorization of enterococci as intrinsically resistant to FA. Similar FA breakpoints were established by Toma and Barriault (1995). The estimated FA susceptibility breakpoint calculated based on the *C*_max_ recorded with the front-loading-dose regimen was ≥ 8 µg/mL. Accordingly, *E. faecalis* ATCC 29212 and *E. faecium* ATCC 27270, as well as all but two of the clinical isolates examined, can be susceptible, suggesting that reconsideration of FA for treatment of enterococci, using the front-loading dose regimen, is warranted.

It is noteworthy to mention that the applied equation for breakpoint estimation is no longer used by the EUCAST or the CLSI (EUCAST 2021; Clinical and Laboratory Standards Institute 2023a). However, it was applied here to estimate the breakpoints in different regimens aiming to determine primarily whether the elevated plasma concentrations attained after applying the front-loading-dose regimen can affect the categorization of enterococci susceptibility to FA. To establish an applicable breakpoint for susceptibility testing by CLSI or EUCAST, data about the dosage regimen, MIC distribution, pharmacokinetics/pharmacodynamics of the agent, modeling as Monte Carlo simulations, and clinical trials outcomes should be available (EUCAST 2021; Clinical and Laboratory Standards Institute 2023). Therefore, based on our results, it is highly recommended to reconsider the calculation of FA susceptibility breakpoints.

To confirm the susceptibility of enterococci to FA, protection against *E. faecalis* infection was evaluated in a BALB/c mouse model. Administration of FA in mice is challenging due to the drug’s rapid metabolism and short half-life, necessitating multiple doses to achieve higher accumulation. Contrary to Payne et al. (2013), who reported that BALB/c mice cannot tolerate more than 20 mg/kg/day of FA q8h, intraperitoneal administration of 60 mg/kg body weight q8h was well-tolerated in the current study. FA administration successfully reduced the bacterial burden of both vancomycin-susceptible and -resistant *E. faecalis* in the liver and spleen by 2–3 log_10_ units compared to untreated controls.

Use of FA in the treatment of *S. aureus* infections is usually associated with resistance development that necessitates coadministration with other antimicrobials (Howden and Grayson 2006; Hajikhani et al. 2021). In enterococci, the transmissible genes (*fus*B, *fus*C, and *fus*D) responsible for FA resistance were not detectable in the publicly available genomes evaluated in this study, although transmission of antimicrobial resistance genes was reported previously between different microbial genera and species (Lerminiaux and Cameron 2019; Li et al. 2023). This excludes the possibility of transfer of FA resistance to enterococci, which is further confirmed by failure to detect any enterococcal strain with FA MIC exceeding 8 µg/mL.

In this study, exposure of enterococci to fixed FA concentrations did not result in resistance development, whereas exposure to increasing concentrations caused an unstable resistance phenotype with a six- to seven-fold increase in MIC towards FA. The susceptible phenotype was restored after passage of the developed resistant strains in a FA-free medium. This corresponds to adaptive resistance according to the definition of Fernández and Hancock (2012). Development of resistance by exposure to increasing FA concentrations was reported previously in *S. aureus* isolates (Abdelkareem et al. 2017).

The developed resistant phenotypes had significantly slower growth rates and thicker cell walls compared to the parent or revertant strains. The slow growth was reported previously in the FA resistant *S. aureus* strain (Gupta et al. 2022). In the present study, the reported cell wall thickening can account for the developed unstable resistance and the slower growth rate as an adaptation to FA exposure. *S. aureus* cell wall thickening was a morphological change associated with antimicrobials acting through inhibition of protein synthesis (Cushnie et al. 2016), and cell wall thickening was observed in daptomycin-resistant enterococci (Miller et al. 2019), methicillin-resistant *S. aureus* (Nakamura et al. 2021), and vancomycin-resistant *S. aureus* (Cui et al. 2003). Cell wall thickening in our study can account for the failure to extract DNA from the resistant strains by the boiling method, similar to the difficulty of extracting the DNA from Gram-positive bacteria compared to Gram-negative ones (Gram-positive bacteria have thicker cell wall) which necessitates using lysis enzymes for the extraction from the Gram-positive bacteria (De et al. 2010). On the other hand, the reported cell wall thickness was not enough to impart resistance to other antimicrobial agents as indicated by the maintained susceptibility to the tested antimicrobials.

No detectable mutations in *fus*A or *fus*E gene sequences or their translated peptides were observed in the resistant or revertant enterococci strains, except one amino acid change (M651I) that was reported in the translated peptide of the *fus*A gene from the resistant *E. faecium* iP15 (MIC = 128 µg/mL) and maintained in the revertant *E. faecium* iP15f (MIC = 4 µg/mL). Loss of the resistance phenotype despite the maintenance of the mutation suggests another adaptation mechanism than the *fus*A gene mutation, which may be the reported thickened cell wall or other untested mutation. The effect of substitution of isoleucine for methionine varied according to the tested proteins; some proteins topologies and activity were affected (Cama et al. 1992), and some were not (Bordo and Argos 1991; Ohmura et al. 2001). However, in our study, it may be a matter of not only the effect on protein topology but also the possible effect of this substitution on the interaction of the protein with FA.

Unlike staphylococci, the reported lack of development of acquired mutational resistance after exposure to increasing FA concentrations may be accounted for by the occurrence of lethal mutations. Bourgogne and colleagues (Bourgogne et al. 2008), reported one amino acid mutation; H404Q in the *E. faecalis* OG1RF strain (FA resistant). Whether *E. faecalis* can develop stable resistance by exposure to FA still needs to be studied more extensively; however, it seems that this does not occur at a high rate.

Further studies are needed to confirm the clinical value of FA for treating patients with an enterococcal infection and, consequently, a re-evaluation of FA breakpoints for pathogens belonging to this genus.

## Conclusions

FA has the potential to be repurposed as an antimicrobial agent against enterococci, including vancomycin-resistant strains, with a low probability of mutational resistance development. This will present an additional option to patients infected with vancomycin-resistant enterococci, for which therapeutic modalities are limited.

## Supplementary Information

Below is the link to the electronic supplementary material.

Supplementary file1 (PDF 423 KB)

Supplementary file2 (XLSX 14 KB)

## Data Availability

All data generated or analyzed during this study are included in this published article and its supplementary information files.
