# Supplementary material for: Repurposing fusidic acid as an antimicrobial against enterococci with a low probability of resistance development
Source: Int Microbiol. 2024 Mar 27;27(6):1807–19. doi: 10.1007/s10123-024-00506-w (PMC11611940; doi:10.1007/s10123-024-00506-w)
Supplement: Supplementary file 1 — Supplementary file1 (PDF 423 KB) [file 10123_2024_506_MOESM1_ESM.pdf]

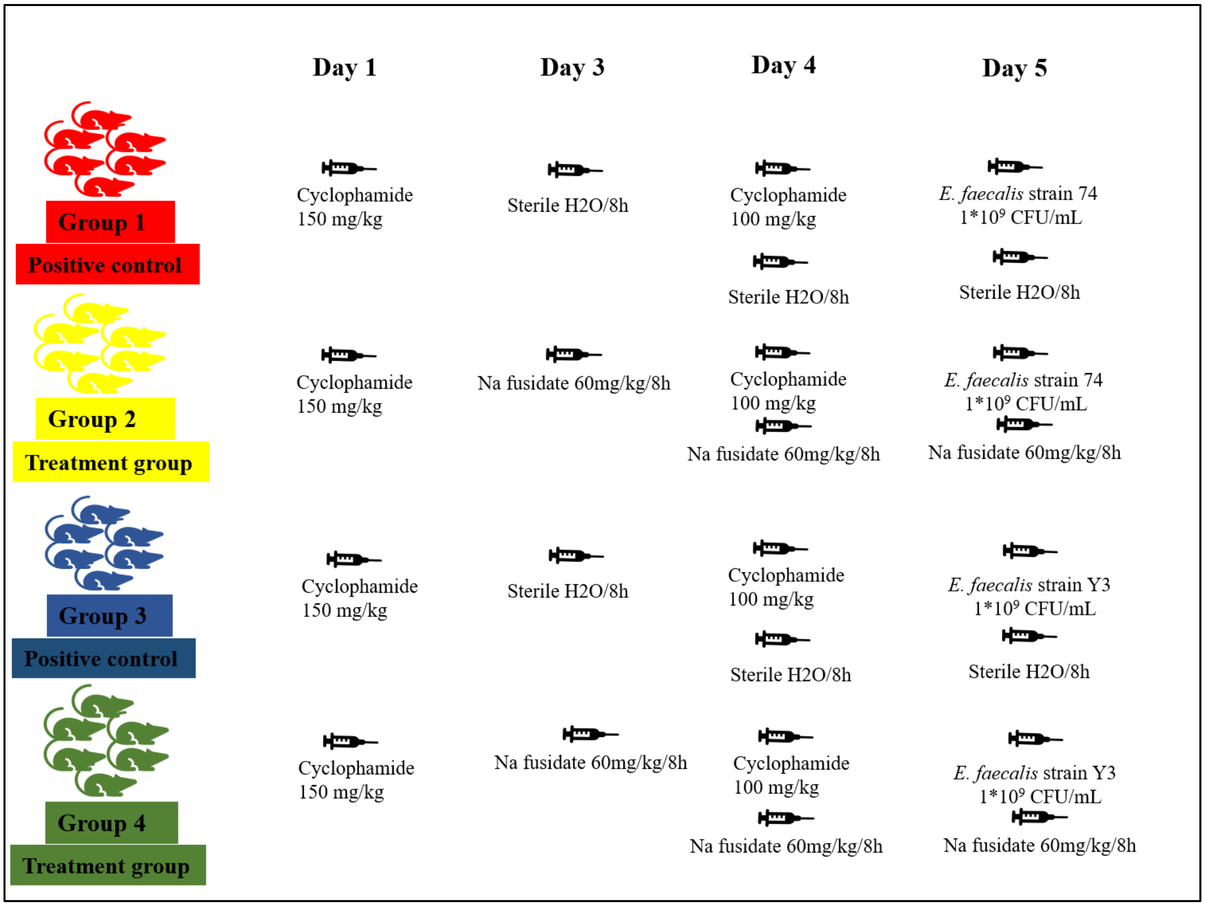

Figure S1 BALB/c mice infection with *E. faecalis* and the applied fusidic acid treatment regimen

Table S1. Accession numbers of the *E. faecium* sequences used in primer design

Strain name	Accession number
Enterococcus faecium DO	CP003583.1
Enterococcus faecium strain VB3025	CP040236.1
Enterococcus faecium strain F17E0263	CP040849.1
Enterococcus faecium strain SRR24	CP038996.1
Enterococcus faecium strain UAMSEF_09	CP035660.1
Enterococcus faecium strain UAMSEF_20	CP035666.1
Enterococcus faecium strain UAMSEF_08	CP035654.1
Enterococcus faecium strain VB3240	CP040368.1
Enterococcus faecium strain RBWH1	CP033206.1

```
E.faecium_ATCC27270    MTREFSLQNTRNIGIMAHIDAGKTTATERILYYTGKIHKIGETHEGASQMDWMEQEQERG 60
E.faecium_ip15         MTREFSLQNTRNIGIMAHIDAGKTTATERILYYTGKIHKIGETHEGASQMDWMEQEQERG 60
E.faecium_ip15f        MTREFSLQNTRNIGIMAHIDAGKTTATERILYYTGKIHKIGETHEGASQMDWMEQEQERG 60
                       ************************************************************

E.faecium_ATCC27270    ITITSAATTAQWKGHRINIIDTPGHVDFTVEVERSLRVLDGAVTVLDAQSGVEPQTETVW 120
E.faecium_ip15         ITITSAATTAQWKGHRINIIDTPGHVDFTVEVERSLRVLDGAVTVLDAQSGVEPQTETVW 120
E.faecium_ip15f        ITITSAATTAQWKGHRINIIDTPGHVDFTVEVERSLRVLDGAVTVLDAQSGVEPQTETVW 120
                       ************************************************************

E.faecium_ATCC27270    RQATTYGVPRIVFANKMDKIGADFLYSVSTLHDRLQANAHPIQLPIGAEDDFTGIIDLVT 180
E.faecium_ip15         RQATTYGVPRIVFANKMDKIGADFLYSVSTLHDRLQANAHPIQLPIGAEDDFTGIIDLVT 180
E.faecium_ip15f        RQATTYGVPRIVFANKMDKIGADFLYSVSTLHDRLQANAHPIQLPIGAEDDFTGIIDLVT 180
                       ************************************************************

E.faecium_ATCC27270    MKAEMYTNDLGTEIEETEIPEEYRELAEEWREKLVEAVAETDEELTLKYLEGEEITEAEL 240
E.faecium_ip15         MKAEMYTNDLGTEIEETEIPEEYRELAEEWREKLVEAVAETDEELTLKYLEGEEITEAEL 240
E.faecium_ip15f        MKAEMYTNDLGTEIEETEIPEEYRELAEEWREKLVEAVAETDEELTLKYLEGEEITEAEL 240
                       ************************************************************

E.faecium_ATCC27270    KEGIRRATVNVEFYPVLCGSAFKNKGVQLLLDAVLDYLPSPLDIPAIKGIDPKTDEEVER 300
E.faecium_ip15         KEGIRRATVNVEFYPVLCGSAFKNKGVQLLLDAVLDYLPSPLDIPAIKGIDPKTDEEVER 300
E.faecium_ip15f        KEGIRRATVNVEFYPVLCGSAFKNKGVQLLLDAVLDYLPSPLDIPAIKGIDPKTDEEVER 300
                       ************************************************************

E.faecium_ATCC27270    PADDSAPFSALAFKVMTDPFVGRLTFFRVYSGVLKSGSYVQNATKGKRERVGRILQMHAN 360
E.faecium_ip15         PADDSAPFSALAFKVMTDPFVGRLTFFRVYSGVLKSGSYVQNATKGKRERVGRILQMHAN 360
E.faecium_ip15f        PADDSAPFSALAFKVMTDPFVGRLTFFRVYSGVLKSGSYVQNATKGKRERVGRILQMHAN 360
                       ************************************************************

E.faecium_ATCC27270    SRSEISEVYAGDIAAAVGLKDTTTGDTLCDEKNLVILESMEFPEPVIQVAIEPKSKADQD 420
E.faecium_ip15         SRSEISEVYAGDIAAAVGLKDTTTGDTLCDEKNLVILESMEFPEPVIQVAIEPKSKADQD 420
E.faecium_ip15f        SRSEISEVYAGDIAAAVGLKDTTTGDTLCDEKNLVILESMEFPEPVIQVAIEPKSKADQD 420
                       ************************************************************

E.faecium_ATCC27270    KMGVALQKLSEEDPTFRAETNVETGETIIAGMGELHLDIIVDRMRREFKVEANVGAPQVS 480
E.faecium_ip15         KMGVALQKLSEEDPTFRAETNVETGETIIAGMGELHLDIIVDRMRREFKVEANVGAPQVS 480
E.faecium_ip15f        KMGVALQKLSEEDPTFRAETNVETGETIIAGMGELHLDIIVDRMRREFKVEANVGAPQVS 480
                       ************************************************************

E.faecium_ATCC27270    YRETFRAGTQAEGKFVRQSGGKGQYGHVWIEFTPNEEGAGFEFENAIVGGVVPREYIPAV 540
E.faecium_ip15         YRETFRAGTQAEGKFVRQSGGKGQYGHVWIEFTPNEEGAGFEFENAIVGGVVPREYIPAV 540
E.faecium_ip15f        YRETFRAGTQAEGKFVRQSGGKGQYGHVWIEFTPNEEGAGFEFENAIVGGVVPREYIPAV 540
                       ************************************************************

E.faecium_ATCC27270    ETGLKDAMENGVLAGYPLVDIKAKLYDGSYHDVDSNETAFRVAASMALRAAAKKANPVIL 600
E.faecium_ip15         ETGLKDAMENGVLAGYPLVDIKAKLYDGSYHDVDSNETAFRVAASMALRAAAKKANPVIL 600
E.faecium_ip15f        ETGLKDAMENGVLAGYPLVDIKAKLYDGSYHDVDSNETAFRVAASMALRAAAKKANPVIL 600
                       ************************************************************

E.faecium_ATCC27270    EPIMAVEVVIPEDYLGDVMGHVTARRGRVEGMEARAGGQQVVRAMVPLA[illegible]M[illegible]GYATTLRS 660
E.faecium_ip15         EPIMAVEVVIPEDYLGDVMGHVTARRGRVEGMEARAGGQQVVRAMVPLA[illegible]I[illegible]GYATTLRS 660
E.faecium_ip15f        EPIMAVEVVIPEDYLGDVMGHVTARRGRVEGMEARAGGQQVVRAMVPLA[illegible]I[illegible]GYATTLRS 660
                       ************************************************* : ********

E.faecium_ATCC27270    ATQGRGTFTMTFDHYEDVPKSVQEEIIKKNGGKAE     695
E.faecium_ip15         ATQGRGTFTMTFDHYEDVPKSVQEEIIKKNGGKAE     695
E.faecium_ip15f        ATQGRGTFTMTFDHYEDVPKSVQEEIIKKNGGKAE     695
                       ***********************************
```

Figure S2 Multiple sequence alignment of the translated sequences of the *fus*A gene from the parent *Enterococcus faecium* ATCC 27270, the resistant *E. faecium* iP15 obtained after the passage of the *E. faecium* ATCC 27270 in increasing fusidic acid concentrations for 15 consecutive passages and the revertant *E. faecium* iP15f obtained after the passage of *E. faecium* iP15 in a fusidic acid-free medium for 10 consecutive passages
